# Graph Neural Network Learning on the Pediatric Structural Connectome

**DOI:** 10.3390/tomography11020014

**Published:** 2025-01-29

**Authors:** Anand Srinivasan, Rajikha Raja, John O. Glass, Melissa M. Hudson, Noah D. Sabin, Kevin R. Krull, Wilburn E. Reddick

**Affiliations:** 1 Departments of Radiology, St. Jude Children’s Research Hospital, Memphis, TN 38105, USA; anand.srinivasan@yale.edu (A.S.); rajikha.raja@stjude.org (R.R.); john.glass@stjude.org (J.O.G.); noah.sabin@stjude.org (N.D.S.); 2 Department of Oncology, St. Jude Children’s Research Hospital, Memphis, TN 38105, USA; melissa.hudson@stjude.org; 3 Department of Psychology and Behavioral Sciences, St. Jude Children’s Research Hospital, Memphis, TN 38105, USA; kevin.krull@stjude.org

**Keywords:** graph neural networks, structural brain connectomes, data enrichment

## Abstract

Purpose: Sex classification is a major benchmark of previous work in learning on the structural connectome, a naturally occurring brain graph that has proven useful for studying cognitive function and impairment. While graph neural networks (GNNs), specifically graph convolutional networks (GCNs), have gained popularity lately for their effectiveness in learning on graph data, achieving strong performance in adult sex classification tasks, their application to pediatric populations remains unexplored. We seek to characterize the capacity for GNN models to learn connectomic patterns on pediatric data through an exploration of training techniques and architectural design choices. Methods: Two datasets comprising an adult BRIGHT dataset (N = 147 Hodgkin’s lymphoma survivors and N = 162 age similar controls) and a pediatric Human Connectome Project in Development (HCP-D) dataset (N = 135 healthy subjects) were utilized. Two GNN models (GCN simple and GCN residual), a deep neural network (multi-layer perceptron), and two standard machine learning models (random forest and support vector machine) were trained. Architecture exploration experiments were conducted to evaluate the impact of network depth, pooling techniques, and skip connections on the ability of GNN models to capture connectomic patterns. Models were assessed across a range of metrics including accuracy, AUC score, and adversarial robustness. Results: GNNs outperformed other models across both populations. Notably, adult GNN models achieved 85.1% accuracy in sex classification on unseen adult participants, consistent with prior studies. The extension of the adult models to the pediatric dataset and training on the smaller pediatric dataset were sub-optimal in their performance. Using adult data to augment pediatric models, the best GNN achieved comparable accuracy across unseen pediatric (83.0%) and adult (81.3%) participants. Adversarial sensitivity experiments showed that the simple GCN remained the most robust to perturbations, followed by the multi-layer perceptron and the residual GCN. Conclusions: These findings underscore the potential of GNNs in advancing our understanding of sex-specific neurological development and disorders and highlight the importance of data augmentation in overcoming challenges associated with small pediatric datasets. Further, they highlight relevant tradeoffs in the design landscape of connectomic GNNs. For example, while the simpler GNN model tested exhibits marginally worse accuracy and AUC scores in comparison to the more complex residual GNN, it demonstrates a higher degree of adversarial robustness.

## 1. Introduction

The human structural connectome represents an individual’s brain connectivity network by quantifying the white matter tracts connecting anatomical gray matter regions in the brain using diffusion-weighted magnetic resonance imaging (DW-MRI). Structural connectome data has been a valuable tool for linking brain structure to neurological function and dysfunction [1]. Specifically, it has enhanced our understanding of typical brain development [2,3] as well as various neurological disorders, including epilepsy [4,5,6], schizophrenia [7,8], Alzheimer’s disease [9,10,11,12], and autism [12,13].

Due to its promising clinical relevance, the structural connectome has been the focus of multiple machine learning studies [14,15,16]. Among these, structural connectome classification stands out as a clinically important task, as classification algorithms could facilitate early diagnosis of neurological disorders and improve our understanding of relevant clinical characteristics at a neuroanatomical level. Such analysis employs graph theory, where the structural connectome represents a brain graph G = (V, E), in which brain regions make up the set of vertices V, and tract connections between these regions make up the set of weighted edges E (with the number of tracts between two regions determining the relative weight of the corresponding edge).

Although the structural connectome includes many descriptive cerebral features, its inherent graph structure poses challenges for traditional learning methods. Conventional machine learning and deep learning approaches, which are designed for Euclidean data like text and images, struggle with non-Euclidean graph data due to their inability to capture a graph’s topological structure [17]. Graph neural networks (GNNs) were developed to address these shortcomings [18]. GNNs are capable of learning and preserving topological patterns, enabling the training of generalizable, high-performance models on graph data [19].

Sex classification has been a major focal point of previous brain connectivity research [20,21,22]. Differentiating sex in the structural connectome may lead to a better understanding of neurological disorders with sex-specific presentations. For example, studies have shown sex-specific brain connectivity patterns in patients with autism [23], mild cognitive impairment [24], and conduct disorder [25], which could help explain the differing presentations of these disorders across sexes. Furthermore, studies show differing developmental brain connectivity patterns between male and female adolescents [22,26]. Developing connectivity-based sex prediction models for pediatric patients therefore could improve the understanding of brain development in children and aid in identifying risk factors and early diagnosis of certain neurological disorders [20]. Moreover, since early machine learning works on the structural connectome focused on sex and standard demographic information, this information is routinely available in most publicly available datasets. Sex classification also represents an intuitive classification benchmark for assessing how models perform across structural connectome datasets [27].

While GNN models have achieved reasonable success in classifying sex in adult patients [20,27], this work has not been extended to pediatric populations. Pediatric patients exhibit distinct brain connectivity patterns, particularly in developing white matter tracts, which form the edges of the structural connectome [3,28,29]. Consequently, models trained on adult structural connectome data may not be directly applicable to pediatric patients. Moreover, the typically smaller size of pediatric datasets presents additional challenges in training effective pediatric models.

One approach to address this problem of small sample sizes in pediatric datasets is to use few-shot leaning, which learns to make accurate classifications by training on a very small number of labeled cases when suitable training data is scarce. Few-shot learning approaches for GNNs in connectomics are not well studied [19]. Here, as a few-shot learning approach, a small pediatric dataset was enriched with a much larger adult data to test if GNN models were able to leverage this additional connectomic data to learn to make more accurate predictions.

In this study, GNNs and other standard machine learning models were trained on whole brain structural connectome data for sex classification of both pediatric and adult participants. Classification accuracy for the larger adult dataset was compared to previous studies to compare the performance of the learning models [20,27]. Trained adult, pediatric, and enriched adult/pediatric models were tested on the pediatric dataset [20].

After exploring training approaches using two selected GNN architectures and three other deep and machine learning models for comparison, a series of GNN architecture exploration experiments were conducted to determine the impact of pooling and aggregation function, model depth, and skip connections on the ability for GNNs to capture connectomic patterns. Further, adversarial robustness experiments were conducted to assess how models responded to adversarial attacks. These experiments sought to characterize the landscape of design choices and performance tradeoffs for connectomic models.

The primary objectives of this study are to evaluate whether an enriched training approach enables the GNN to generalize across age groups, achieving pediatric classification accuracy comparable to that of adult GNN models tested on adult datasets and to explore the impact of GNN architectural design choices on connectomic learning ability.

## 2. Materials and Methods

### 2.1. Datasets

The Human Connectome Project in Development (HCP-D) sought to characterize brain connectivity over the course of typical development. The pediatric HCP-D dataset included 135 healthy participants, ranging from 8 to 20 years of age with a mean age of 16 years. Participants with health conditions that might have impacted typical development or jeopardized their inclusion within the dataset were excluded. 3D T1 weighted images and DW-MRI sequences were acquired with 3T Siemens Prisma scanners in two shells of b = 1500 and 3000 s/mm^2^ along 185 gradient directions [3].

The BRIGHT dataset included 147 adult survivors of childhood Hodgkin’s lymphoma (HL) and 162 control participants recruited to frequency match survivors across sex, age, and race/ethnicity. All survivors were 18 years of age or older with a mean age of 36 years. All survivors received thoracic radiation during initial treatment and were 5 or more years from diagnosis at the time of data collection. 3D T1 weighted images and DW-MRI sequences were acquired with a 3T scanner in a single shell of b = 700 s/mm^2^ along 30 gradient directions. Demographics from both cohorts are reported in Table 1.

### 2.2. Structural Connectome Processing

The processing pipeline includes multiple workflows implemented in succession. First, raw DW-MRI data were corrected for noise, motion, and Eddy currents [30]. Images were then analyzed using a spherical deconvolution diffusion model to generate fiber orientation distribution functions (FOD) and probabilistic tractography was performed to attain whole brain tractograms using the MRtrix3 (https://www.mrtrix.org (accessed on 12 March 2023)) iFOD2 algorithm [31]. The whole brain streamlines were filtered using the SIFT2 method to extract realistic streamlines [32]. Whole brain structural connectomes were computed for each subject based on weighted streamline density from the whole brain tractograms and based on the parcellations in the HCP-MMP1.0 atlas consisting of 379 cortical and sub-cortical regions as nodes [33].

### 2.3. Graph Convolutional Network (GCN) Model Definition

Let a dataset D of size N be defined as $D=\left(D_{1},D_{2},\ldots ,D_{N}\right)$. The *i*-th patient in dataset D can be described as $D_{i}=\left(G_{i},X_{i},y_{i}\right)$, where ${G_{i}=(V}_{i},E_{i})$ is the patient’s structural connectivity graph with its corresponding node feature matrix $X_{i}\in R^{\left|V_{i}\right|\times d}$ and class label $y_{i}=\left\{0,1\right\}$. Any patient graph $G_{i}$ can also be represented as an adjacency matrix $A_{i}\in R^{\left|V_{i}|\times |V_{i}\right|}$. A model function $f_{\theta }:{(G}_{i},X_{i})\to {\hat{y}}_{i}$ was defined where θ is the set of learnable parameters and ${\hat{y}}_{i}$ is the model’s predicted class label. The objective was to learn a function f which minimizes the classification loss over the dataset D.

This study focuses on GNN models, specifically Graph Convolutional Networks (GCNs). GCNs aim to create s-dimensional node embeddings for each node in a graph $G_{i}$. These individual node embeddings can subsequently be pooled into a single s-dimensional graph embedding vector, which can finally be fed into a downstream classifier for prediction of the output label. GCNs generate node embeddings through an iterative, message passing algorithm. Nodes send messages to their surrounding neighborhoods, and these messages are aggregated and used to update each node’s current embedding.

A single layer of a GCN model can be defined as:

(1) $$H_{i}^{l}=\sigma \left(A_{i}^{*}H_{i}^{\left(l-1\right)}W+b\right)$$

(2) $$H_{i}^{0}=X_{i}$$

where $A_{i}^{*}$ is the normalized adjacency matrix representation of the patient graph $G_{i}$, which is derived from structured connectome data. $A_{i}^{*}$ is a symmetric matrix with dimensions corresponding to the number of nodes in the graph. In this study, we utilized 379 nodes defined by the HCP-MMP1.0 atlas for the whole brain, resulting in $A_{i}^{*}$ being a 379 × 379 matrix. $H_{i}^{\left(l-1\right)}$ is the node embedding matrix from the previous iteration, W is the learnable weight matrix, b is the learnable bias term, σ is the non-linear activation function. In this study, we use the ReLU (Rectified Linear Unit) and tanh activation functions for σ. $H_{i}^{l}$ is the resulting node embedding matrix of the GCN layer at the *i*-th iteration. The node embedding matrix is initialized with the node feature matrix in the first iteration. In the above equation, $H_{i}^{\left(l-1\right)}W$ represents the messages passed from each node to its surrounding neighborhood, and $A_{i}^{*}H_{i}^{\left(l-1\right)}W$ represents the weighted aggregation of these outgoing messages based upon the strength of the connection between nodes in the normalized adjacency matrix. Individual GCN layers can be stacked or joined via skip connections to build larger networks.

### 2.4. Model Architecture

**Figure 1:**
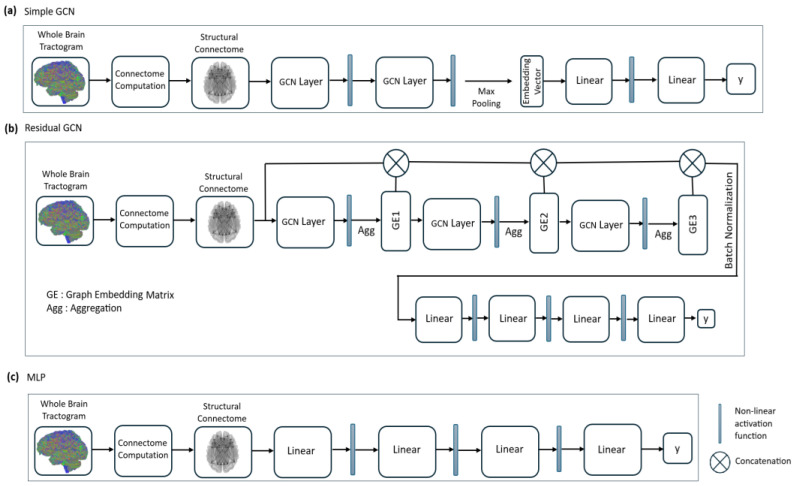
Model architecture illustrations for simple GCN (**a**), residual GCN (**b**), and multi-layer perceptron (**c**) networks.

Two GCN models (simple [34] and residual), a deep neural network (multi-layer perceptron (MLP)), and two standard machine learning models (random forest (RF) and support vector machine (SVM)) were trained. Random Forest and SVM were selected as baseline models to enable a meaningful comparison with GNNs. Random Forest was chosen for its resilience to noise, capability to manage high-dimensional data like structural connectome features. SVM was included for its proven effectiveness in binary classification tasks and its capacity to capture non-linear relationships through kernel methods. Unlike GNNs, which directly utilize the graph structure of the connectome, these models rely on predefined features, offering a contrasting perspective. This comparison allowed us to highlight the benefits of graph-based learning and demonstrate the superior performance of GNNs in this context. Model architectures for both GCN models and the MLP are shown in Figure 1, and model sizes for these models are displayed in Table 2. The simple GCN model contains GCN layers with 64 neurons each, while the residual GCN model contains GCN layers with 32 neurons each. The simple GCN model uses ReLU activation functions between graph convolutional layers and mean global pooling for the generation of a graph embedding vector, while the residual GCN model uses tanh activation and mean aggregation functions between graph convolutional layers to create intermediate graph embedding matrices. The model architecture for the residual GCN is adapted from a model proposed in a brain connectomics benchmarking paper [5]. For the MLP network, hidden layers with 512, 256, and 128 neurons are used with ReLU activation. All models were implemented with PyTorch Geometric (version 2.7.0).

### 2.5. Training Procedure

The hyperparameter space for all deep learning models was explored extensively. All deep learning models trained with a learning rate of 1 × 10^−3^ for 100 epochs using weighted binary cross entropy loss. A number of regularization techniques were employed to mitigate overfitting. 50% dropout was added between GCN layers and fully connected layers for GNN and MLP models, respectively. All deep learning models were trained using a weight decay of 5 × 10^−4^, and early stopping was adopted with a patience of 35 epochs.

For the RF and SVM models, principal component analysis was used to reduce connectivity matrix data to 100 dimensions. The random forest model was initialized with 100 estimators, and the support vector machine applies the radial basis function (rbf) kernel. All other parameters are left as default.

Data was split with 70% for training, 10% for validation, and 20% testing in all deep learning experiments. For the two standard machine learning models (RF and SVM), 80% of the data was used for training and the remaining 20% was used for testing. Five-fold cross validation was employed to generate five unique testing sets. The mean accuracy and AUC scores across all sets is reported.

### 2.6. Model Evaluation Experiments

First, all models were trained and tested against the two available datasets individually. For experiments on individual datasets, data from a single dataset was split into training, validation, and testing sets and each model’s mean accuracy and AUC score on the unseen test data is reported. Models trained on the adult BRIGHT dataset were also externally validated on pediatric HCP-D data to test how well structural connectivity models trained solely on adult participants generalize to pediatric participants.

Finally, models were trained and tested on the adult-enriched pediatric dataset. Data from both datasets was shuffled together and split into training, validation, and testing sets. The ratio of adults and pediatric data was held constant across all sets. Each model’s accuracy on the unseen test data was reported both as an overall score and stratified by the dataset of origin.

### 2.7. GNN Architecture Exploration Experiments

After initial model evaluations, a variety of architectural exploration and ablation experiments were conducted to characterize the impact and effect of different model components on performance.

Mean and max pooling and aggregation approaches were compared to investigate the impact of these pooling and aggregation methods on the ability of the GNN models to learn connectomic patterns. The simple GCN, which initially used a mean global pooling function to generate a graph embedding vector, was tested with a max global pooling function replacement, while the residual GCN, which uses a mean node aggregation for graph feature generation, was tested with a max aggregation function replacement. Both tests were performed using the adult-enriched dataset for training and evaluation.

Next, the effect of model depth was investigated for both GNNs. The simple GNN was initially evaluated with two GCN layers. Deeper analogs with additional GCN layers were tested to determine if increasingly depth would improve the model. The residual GCN was initially evaluated with three GCN layers. Two- and four-layer analogs were tested on the adult-enriched dataset to determine the effect of decreasing or increasing the depth on the residual network.

Finally, an ablation experiment was performed to test how skip connections contributed to the ability of the residual GCN model to learn on connectomic data. All skip connections were removed, and the resulting model was trained and tested on the adult-enriched dataset.

### 2.8. Adversarial Sensitivity Experiments

Adversarial attacks are small, targeted perturbations applied to input data in order to mislead classification models. In the medical domain, robustness to adversarial attacks is important, as errors caused by such attacks could result in harmful or even fatal consequences. Further, a model’s sensitivity to small adversarial perturbations may be an indicator of robustness to general noise, which is an inevitable component of real-world medical data.

Adversarial robustness for all deep learning models was evaluated using the fast gradient sign method (FGSM) adversarial attack. FGSM is a white-box adversarial evasion attack which can be defined as:

(3) $$X_{i}^{adv}=X_{i}+\epsilon *sign(\nabla_{X}L\left(\theta ,X_{i},y_{i}\right))$$

where $X_{i}$ is a node feature matrix corresponding to an input graph $G_{i}$, L is the loss as function of the model’s trainable parameters θ, the node feature matrix $X_{i}$ and the corresponding label $y_{i}$, ϵ is the size of the adversarial perturbation, and $X_{i}^{adv}$ is the resulting perturbed node feature matrix.

White box attacks like FGSM enjoy full access to a model’s loss function and trainable parameters. As such, they can construct powerful, targeted adversarial examples. Increasing the value of epsilon increases the distance between the original graph and the adversarial example, therefore leading to stronger adversarial attacks. All deep learning models trained on the adult-enriched dataset were tested on target adversarial examples generated by the FGSM method for a range of epsilon values. The average resulting accuracy and AUC scores across the five cross validation test sets are reported.

All code used in this study is publicly available: https://github.com/SrinivasanAnand/GNN_structural_connectivity (accessed on 10 December 2024).

## 3. Results

### 3.1. Adult Training and Adult Testing

The two standard machine learning models RF and SVM, the deep learning neural network MLP, and the two GCN models (simple and residual) were trained and tested on the adult BRIGHT dataset. The corresponding accuracy and AUC score for each model is shown in Table 3. The GCN models outperform others on the adult dataset, with accuracies exceeding 82%, followed by the multi-layer perceptron model at 77%. GCN models also demonstrate the best AUC scores, exceeding 0.90 for both models.

### 3.2. Adult Training and Pediatric Testing

All classification models were next trained on the adult BRIGHT dataset and tested on the pediatric HCP-D dataset. Experimental results are shown in Table 4. The residual GCN model remains the most robust, achieving an accuracy above 74% and an AUC score of 0.86. All other models lagged with accuracies of 50–60%. Although the residual GCN exhibits a degree of robustness, all models trained on adult data performed sub-optimally for classification of the pediatric datasets.

### 3.3. Pediatric Training and Pediatric Testing

Given the anticipated sub-optimal performance of the adult models for external validation on the pediatric dataset, all classification models were next trained and tested on the pediatric HCP-D dataset. The corresponding accuracy and AUC score for each model is shown in Table 5. The simple GCN model demonstrated the highest accuracy in the low-70% range, while the residual GCN achieved the highest AUC score of 0.85. The residual GCN shows strong discriminative power in terms of AUC score, but all models achieve sub-optimal classification accuracy.

### 3.4. Adult-Enriched Pediatric Dataset Training and Testing

Given the sub-optimal performance of the models trained on either the adult or pediatric datasets for classification of the pediatric testing set, an adult-enriched pediatric dataset was then used for both training and testing. Table 6 displays the performance of the models when classifying the HCP-D pediatric data within the adult-enriched pediatric testing dataset. The residual GCN model excels in the pediatric subset of the test set, achieving an accuracy of 83%, and the simple GCN model reaches 79% classification accuracy. These model performances demonstrate a noticeable improvement compared to the next best classifier within this training approach and any other classifier’s performance across all training approaches on the HCP-D pediatric dataset.

**Figure 2:**
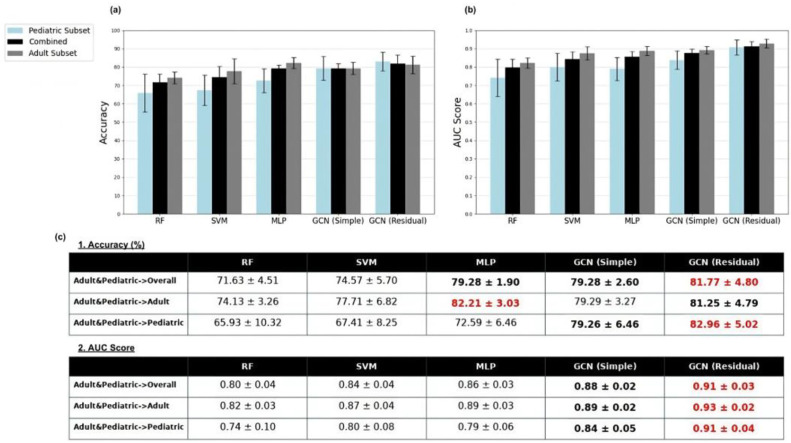
Sex classification mean accuracy (**a**) and AUC score (**b**) results for random forest (RF), support vector machine (SVM), multi-layer perceptron (MLP), simple graph convolutional neural network (GCN simple), and residual graph convolutional neural network (GCN residual) classifiers trained on the adult-enriched pediatric dataset. Results for pediatric (blue), adult (dark gray), and overall (black) subsets of the test dataset were displayed. Standard deviations of classification accuracy shown by whiskers. AUC and accuracy score results are displayed in tabular format in (**c**). Red indicates overall best results; bold indicates overall second-best results.

For completeness, the overall classification accuracies of the models of the adult-enriched pediatric dataset are presented in Figure 2 for the overall testing set and the sub-sets of adult and pediatrics. Overall, the GCN models demonstrate the strongest performance across accuracy and AUC metrics. Notably, the multi-layer perceptron also exhibits strong classification ability under this training approach.

**Figure 3:**
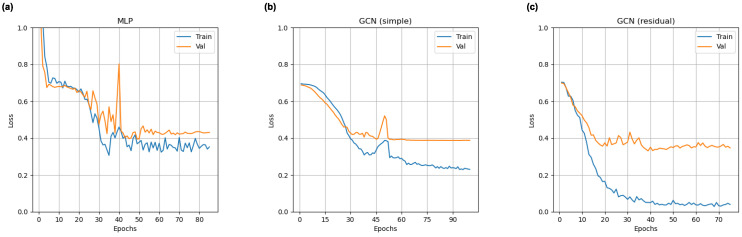
Representative training and validation loss curves for multi-layer perceptron (**a**), simple graph convolutional neural network (**b**), and residual graph convolutional neural network (**c**) classifiers trained on the adult-enriched pediatric dataset.

Loss curves for all deep learning models from a representative cross validation split for this training approach are shown in Figure 3. All models exhibit an ability to learn connectomic features. Both GCNs show signs of overfitting, but this trend is more pronounced in the residual model.

**Figure 4:**
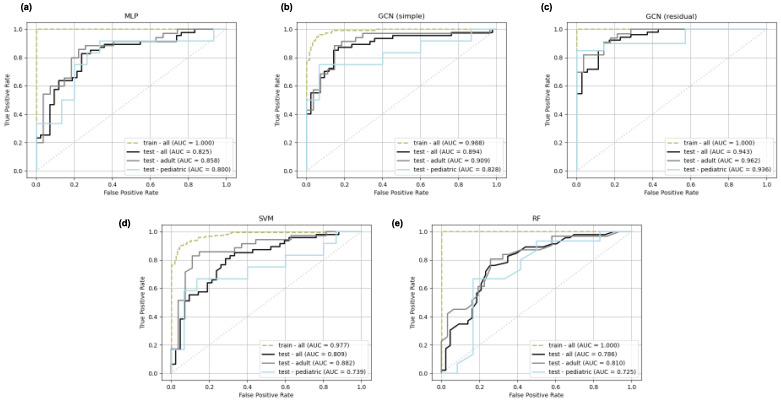
Representative receiver operating characteristic (ROC) curves for multi-layer perceptron (**a**), simple graph convolutional neural network (**b**), residual graph convolutional neural network (**c**), support vector machine (**d**), and random forest (**e**) classifiers trained on the adult-enriched pediatric dataset. Three test set ROC curves are displayed for each model corresponding to pediatric (blue), adult (gray), and overall (black) subsets. A single ROC curve corresponding to the overall training set (green) is displayed for each model.

Receiver operating characteristic (ROC) curves on the training and test set for all models from a representative cross validation split are depicted in Figure 4. Three ROC curves are displayed for each test set, corresponding to the overall test set and the pediatric and adult stratifications. As follows from the AUC score results, the GCN models demonstrate the strongest ROC curves.

### 3.5. GNN Architecture Exploration Results

Architecture exploration experiments were conducted in order to determine the impact of architectural design choices on the ability of the GNN models to learn connectomic patterns. Since the focus of this study is the pediatric connectome, pediatric classification accuracy and AUC scores were used as the primary measure of model performance for exploration experiments. All models were trained on the adult-enriched pediatric dataset since this training method was previously determined to produce the strongest models.

Max and mean pooling and aggregation types were compared across the two GCN models. Table 7 and Table 8 display accuracy and AUC scores for the simple and residual GCN models trained with mean and max pooling and aggregation approaches on the combined dataset. The choice of pooling or aggregation type does not show any major effect.

**Figure 5:**
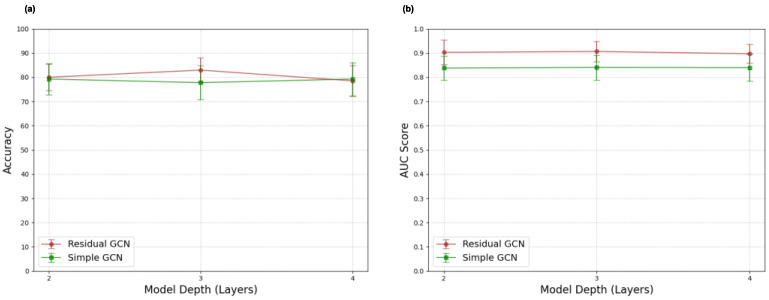
Sex classification mean accuracy (**a**) and AUC score (**b**) results for simple graph convolutional neural network (simple GCN) and residual graph convolutional neural network (residual GCN) architectures with varying model depth analogs.

Simple and residual GCN models of varying depth were tested to assess the impact of depth on learning ability. Results for depth experiments are presented in Figure 5. Again, no major effect is observed, although it is noteworthy that the 3-layer residual network outperforms its 2-layer and 4-layer analogs by 3 accuracy points. For the simple GCN model architecture, shallow networks perform just as well as deeper ones.

Skip ablation experimental results are shown in Table 9. Removing skip connections from the residual GCN network results in a large deterioration in performance, with a 14-point accuracy drop on the pediatric stratification of the dataset, highlighting the importance of skip connections for allowing the residual network to learn connectomic patterns.

### 3.6. Adversarial Sensitivity Experiment Results

**Figure 6:**
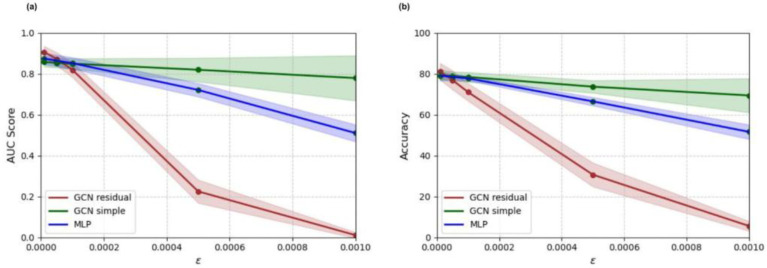
Adversarial accuracy (**a**) and AUC score (**b**) results for multi-layer perceptron (MLP), simple graph convolutional neural network (GCN simple), and residual graph convolutional neural network (GCN residual) classifiers trained on the adult-enriched pediatric dataset.

All deep learning models trained on the adult-enriched dataset were tested on adversarial test sets, generated by targeted FGSM attacks on the pediatric stratification of the enriched test dataset. Perturbation sizes between 1.0 × 10^−5^ and 1.0 × 10^−3^ were tested. Accuracy and AUC score results for all deep learning models on perturbed examples are shown in Figure 6. The simple GCN model remains the most robust to adversarial attacks, followed by the multi-layer perceptron and the residual GCN, respectively.

## 4. Discussion

Experimental results on the adult BRIGHT dataset indicated that graph neural networks outperform traditional machine learning and deep learning approaches in classifying adult structural connectome data. The highest classification accuracy (85.1%) was achieved by the simple GCN model, which surpasses the best non-GCN model by 7.5%. These findings were consistent with the 86.7% reported in previous studies [20,27].

To assess the robustness of the models trained on the adult dataset, external validation was conducted using the pediatric dataset, allowing us to evaluate the generalizability of these models across different patient age demographics. The results reveal that most models were not robust to this demographic shift, an outcome that was anticipated given the inherent variability between the two datasets. The adult dataset, comprising both HL survivors and community controls, used a slightly different diffusion imaging protocol compared to the pediatric dataset, which included only healthy controls. Moreover, it is well-documented that adults exhibit distinct structural connectivity patterns compared to neurologically developing children and teenagers [3,22,26]. Given these distinctions, it is unsurprising that most models trained on the adult BRIGHT dataset struggled to generalize the HCP-D data.

However, it is noteworthy that the residual GCN model exhibited a degree of robustness in the external validation, maintaining an accuracy of over 74% on the pediatric dataset. This performance suggests that the residual GCN model could capture highly generalizable patterns within graph data, extending applicability from adult participants to their pediatric counterparts. Consequently, robust adult-trained graph neural network connectome models may hold potential for direct application to pediatric populations in future clinical settings. This observed generalizability could be particularly valuable in extending the benefits of future connectome-based algorithms to pediatric participants, especially given that the limited size of pediatric datasets often constrains the development of models trained exclusively on pediatric data.

Experiments on the pediatric HCP-D dataset demonstrated that all models were unable to learn highly generalizable patterns on the pediatric connectome without any form of data enrichment. The best model tested was the simple GCN model, which achieves a classification accuracy of 71.1%. The 14-point performance gap observed between the top-performing adult and pediatric models is likely attributable to the smaller size of the pediatric dataset, a limitation that subsequent experiments aimed to address.

An adult-enriched pediatric dataset training approach was employed to assess whether this method could yield stronger models for pediatric participants. This approach functions as a form of data enrichment, utilizing adult patient data to enhance the pediatric dataset and thereby facilitate the training of more robust pediatric models. Additionally, a subset of the adult data was reserved to enable testing of each model’s performance on both unseen pediatric and adult datasets. The enrichment approach allowed deep learning models to achieve strong classification performance, with the residual GCN reaching 81.8% and the simple GCN and multi-layer perceptron both following at 79.3%. Notably, the residual GCN model also performed exceptionally well across both pediatric and adult participants, attaining an accuracy of 83.0% and 81.3%, respectively. The pediatric test accuracy of the residual GCN model is higher than that of all other pediatric models across all training approaches, narrowing the gap between the best achievable pediatric and adult test accuracies to within about two percentage points (83.0 vs. 85.1%). Thus, enriching smaller pediatric datasets with adult structural connectome data can enhance model performance, enabling the development of pediatric models that achieve performance levels comparable to state-of-the-art adult models. While this study enriches pediatric data using adult data to improve model performance, alternative strategies for addressing the limited availability of pediatric connectome datasets should be explored. One potential approach is to collaborate with other pediatric research institutes or initiatives, which may provide access to larger and more diverse pediatric datasets. Examples include publicly available repositories like the Pediatric Imaging, Neurocognition, and Genetics (PING) study [35] or the ABCD dataset [36], which, although limited in diffusion MRI, could still complement structural connectome analyses. Another promising strategy involves leveraging data augmentation techniques specific to connectome graphs, such as perturbation or simulation of biologically plausible variations. These approaches could mitigate the dependence on adult data enrichment while ensuring that the model remains focused on pediatric specific characteristics.

The limited availability of structural connectome data led to the selection of an adult dataset comprising both HL survivors and community controls, while the pediatric dataset included only healthy controls. These datasets were collected using slightly different DW-MRI protocols including different gradient directions and b-values. Additionally, preprocessing pipelines varied, with adult dataset employing standard motion correction and eddy current correction, while pediatric dataset incorporated advanced susceptibility artifact correction. These variations can impact the derived connectome features, with lower b-values or few-er directions potentially reducing tractography accuracy and voxel resolution differences influencing connectivity matrix representations. Consequently, the observed poor external validation results may reflect these protocol differences rather than a lack of model robustness to the population demographics. While this study focuses on improving model generalizability, future work could benefit from using datasets collected with consistent DW-MRI protocols to better isolate the effects of patient demographics. Alternatively, protocol harmonization techniques, such as advanced preprocessing or domain adaptation methods, could mitigate these challenges and enhance model robustness. Despite these limitations, the inclusion of datasets with differing DW-MRI protocols reflects the practical challenges of applying machine learning models across diverse clinical and research settings. Models that generalize well across such variations are more likely to succeed in real-world applications, making this an important avenue for further investigation. Nevertheless, the residual GCN model’s strong external validation performance suggested its capacity to learn highly generalizable patterns that extend from adult to pediatric participants. These generalizable patterns likely represent biologically relevant and stable features within structural connectomes. For instance, the model appeared to leverage connectivity profiles of key hub regions such as the precuneus, posterior cingulate cortex, and superior frontal gyrus, which are central nodes in the brain’s structural network and play a crucial role in maintaining global communication [37,38,39]. These regions are recognized as stable and central nodes, showing relatively low variability across demographic groups [40,41]. Additionally, strong patterns were reported previously in inter-hemispheric connections, particularly in commissural pathways like the corpus callosum. These connections are highly conserved across individuals and age groups, making them reliable features for model generalization [42,43,44,45,46]. The model likely captured patterns of hierarchical organization, including connectivity differences between primary sensory-motor regions, which mature earlier, and higher-order associative areas, which exhibit prolonged development and greater integration over time. These patterns align with established neurodevelopmental trajectories and demonstrate consistency across age groups [47,48,49,50,51]. These patterns suggest that the residual GCN model leveraged features that are biologically consistent and less variable across demographics, contributing to its generalizability.

The differences between the two datasets may have limited the effectiveness of the adult-enriched pediatric dataset training approach. Ideally, data enrichment should be performed using data that closely resembles the original dataset. The pediatric dataset in this study covered ages 8–20 years, while the adult dataset ranged from 18–65 years with a mean age of 35 years. The overlap between the older pediatric participants (18–20 years) and younger adults (18–20 years) helped bridge the two datasets and reduce discontinuities in connectome patterns. Furthermore, the relatively stable nature of structural connectomes in adulthood supports the use of a broader adult age range [52,53,54]. To minimize potential biases, stratified sampling was used during training and cross-validation to ensure proportional representation of both groups. While these strategies mitigated bias, future work could refine this approach by exploring harmonization methods or focusing on more age-restricted datasets to ensure even greater neurodevelopmental consistency. Additionally, employing an adult dataset composed solely of healthy controls and collected using the same DW-MRI protocol as the pediatric dataset might have further enhanced the models’ performance. Future research should explore the efficacy of the data enrichment approach using other adult and pediatric datasets across a range of tasks to validate its ability to improve pediatric models. Although the enrichment of pediatric datasets using adult structural connectomes improved classification performance, achieving 83% accuracy on pediatric data, further gains might be possible through explicit domain adaptation techniques. Methods such as adversarial domain adaptation, which aligns feature distributions between source (adult) and target (pediatric) domains, could help address inherent differences in brain connectivity patterns between age groups. Additionally, transfer learning approaches, where a model pre-trained on adult data is fine-tuned on pediatric data, could allow the model to learn pediatric-specific features more effectively. These strategies could complement the enrichment approach and enhance the generalizability and robustness of the GNN for pediatric classification tasks.

Graph neural network architecture exploration experiments elucidated the impact of several architectural design choices on model performance and learning ability. Depth experiments revealed that increasing the number of GCN layers did not improve the simple GCN model. A moderate 3% accuracy improvement was observed on the pediatric stratification of the combined test set on increasing the depth of the residual GCN from 2 to 3 layers, but the further addition of a fourth GCN layer led to a performance drop on the pediatric stratification. These results indicate that for connectomic applications, a shallow depth of 2 to 3 GCN layers is sufficient for strong performance.

Architecture experiments also highlighted the importance of skip connections for the residual GCN model’s ability to learn pediatric connectomic patterns. An ablation study removing skip connections resulted in a 14-point performance drop in pediatric classification accuracy. Because the only major distinction between the ablated residual network and the simple GCN model was the use of aggregation versus pooling in order to create an embedded graph representation, these results may suggest that connectomic models which employ node aggregation approaches should introduce skip connections for optimal performance.

In addition to accuracy and AUC score metrics, models were assessed on adversarial robustness. The simple GCN model demonstrated the strongest adversarial robustness to FGSM attacks for larger perturbations. The residual GCN, on the other hand, demonstrated a notable lack of adversarial robustness, falling to below 40% classification accuracy for adversarial attacks with epsilon greater than or equal to 0.0005. The residual GCN model’s lack of robustness may in part be a result of its higher complexity. Previous work has shown that higher complexity models exhibit greater sensitivity to adversarial attacks [55]. Further, training and validation loss curves illustrated that the residual GCN overfitted the training set. It is likely that the residual GCN model learned patterns derived from noise in the training set, making it more susceptible to adversarial attacks. Therefore, although the residual GCN model’s higher complexity likely allowed it to learn more generalizable connectomic patterns than the other tested models, this complexity also resulted in a weakness to adversarial attacks. This potential tradeoff between raw test accuracy and adversarial robustness should be considered during model development, especially in the medical domain, where robustness is a key consideration for deploying trustworthy models.

While structural connectome data has recently been applied in graph classification tasks (e.g., sex classification) and graph prediction tasks (e.g., functional connectome prediction), graph regression tasks such as cognitive score prediction remain largely unexplored. These regression tasks hold potential for linking specific brain structural patterns to cognitive functions, thereby enhancing our understanding of cognitive processes and dysfunctions. Although both datasets selected for this study included measures of working memory and sustained attention for most participants, models developed here were not successful in predicting cognitive scores from structural connectome data. Future research with larger datasets or alternative learning approaches may provide better outcomes in this area.

## 5. Conclusions

This study demonstrated the effectiveness of GNNs in classifying sex based on structural connectome data, particularly in pediatric populations, a domain previously unexplored. Training and evaluating GNNs alongside standard machine learning models confirmed that GNNs outperformed other methods in both adult and pediatric datasets. While adult-trained models exhibit limited applicability to pediatric participants, enriching a pediatric dataset with adult data enables the development of a robust pediatric GNN model with performance comparable to adult models. A complex residual GCN architecture enables the best model performance in terms of classification accuracy and AUC score, while a simpler GCN architecture demonstrates greater adversarial robustness at the expense of a moderate performance drop. These findings underscored the potential of GNNs in advancing our understanding of sex-specific neurological development and disorders, highlighting important tradeoffs in the GNN architectural landscape and underscoring the importance of data enrichment in overcoming challenges associated with small pediatric datasets. Future research should continue to explore the application of GNNs to diverse clinical populations and investigate additional strategies for enhancing model generalizability across demographic groups.

## Figures and Tables

**Table 1:** Summary statistics for adult BRIGHT and pediatric HCP-D datasets.

Dataset	Patient Population	N	% Female
BRIGHT	Adult	309	53.40
HCP-D	Pediatric	135	56.30

**Table 2:** Model sizes for multi-layer perceptron (MLP), simple graph convolutional neural network (GCN simple), and residual graph convolutional neural network (GCN residual).

	MLP	GCN (Simple)	GCN (Residual)
Number of Model Parameters	7.37 × 10^7^	3.89 × 10^4^	4.78 × 10^6^

**Table 3:** Sex classification mean accuracy and AUC score results for random forest (RF), support vector machine (SVM), multi-layer perceptron (MLP), simple graph convolutional neural network (GCN simple), and residual graph convolutional neural network (GCN residual) classifiers trained and tested on the BRIGHT (adult) dataset with errors determined by the sample standard deviation. Red indicates overall best results; bold indicates overall second-best results.

Metric	RF	SVM	MLP	GCN (Simple)	GCN (Residual)
Accuracy (%)	73.13 ± 2.05	76.37 ± 5.84	77.66 ± 3.18	**85.10 ± 2.84**	**82.82 ± 5.30**
AUC Score	0.80 ± 0.01	0.86 ± 0.03	0.89 ± 0.01	**0.91 ± 0.03**	**0.93 ± 0.03**

**Table 4:** Sex classification mean accuracy and AUC score results for random forest (RF), support vector machine (SVM), multi-layer perceptron (MLP), simple graph convolutional neural network (GCN simple), and residual graph convolutional neural network (GCN residual) classifiers trained on the BRIGHT (adult) dataset and tested on the HCP-D (pediatric) dataset with errors determined by the sample standard deviation. Red indicates overall best results; bold indicates overall second-best results.

Metric	RF	SVM	MLP	GCN (Simple)	GCN (Residual)
Accuracy (%)	55.70 ± 3.23	56.15 ± 3.79	53.93 ± 3.85	**60.74 ± 3.47**	**74.96 ± 2.41**
AUC Score	0.62 ± 0.01	0.64 ± 0.03	0.60 ± 0.01	**0.71 ± 0.03**	**0.86 ± 0.03**

**Table 5:** Sex classification mean accuracy and AUC score results for random forest (RF), support vector machine (SVM), multi-layer perceptron (MLP), simple graph convolutional neural network (GCN simple), and residual graph convolutional neural network (GCN residual) classifiers trained and tested on the HCP-D (pediatric) dataset with errors determined by the sample standard deviation. Red indicates overall best results; bold indicates overall second-best results.

Metric	RF	SVM	MLP	GCN (Simple)	GCN (Residual)
Accuracy (%)	**66.67 ± 8.76**	65.93 ± 8.25	56.30 ± 7.55	**71.11 ± 10.84**	**66.67 ± 12.83**
AUC Score	0.73 ± 0.13	**0.80 ± 0.04**	0.67 ± 0.05	0.76 ± 0.09	**0.85 ± 0.05**

**Table 6:** Sex classification mean accuracy and AUC score results for random forest (RF), support vector machine (SVM), multi-layer perceptron (MLP), simple graph convolutional neural network (GCN simple), and residual graph convolutional neural network (GCN residual) classifiers trained on the adult-enriched pediatric dataset and tested on the pediatric subset of the test set with errors determined by the sample standard deviation. Red indicates overall best results; bold indicates overall second-best results.

Metric	RF	SVM	MLP	GCN (Simple)	GCN (Residual)
Accuracy (%)	65.93 ± 10.32	67.41 ± 8.25	72.59 ± 6.46	**79.26 ± 6.46**	**82.96 ± 5.02**
AUC Score	0.74 ± 0.01	0.80 ± 0.08	0.79 ± 0.06	**0.84 ± 0.05**	**0.91 ± 0.04**

**Table 7:** Sex classification mean accuracy and AUC score results simple graph convolutional neural network using mean and max pooling functions for graph embedding vector generation.

Pooling Function	Accuracy (%)	AUC Score
Mean	79.26 ± 6.46	0.91 ± 0.05
Max	77.04 ± 7.18	0.85 ± 0.04

**Table 8:** Sex classification mean accuracy and AUC score results for the residual graph convolutional neural network model using mean and max aggregation functions for graph embedding matrix generation.

Aggregation Function	Accuracy (%)	AUC Score
Mean	82.96 ± 5.02	0.91 ± 0.04
Max	81.48 ± 5.24	0.90 ± 0.04

**Table 9:** Sex classification mean accuracy and AUC score results for the residual graph convolutional neural network with and without skip connections.

Residual GCN Model (With/Without Skips)	Accuracy (%)	AUC Score
Without Skips	68.89 ± 9.54	0.74 ± 0.07
With Skips	82.96 ± 5.02	0.91 ± 0.04

## Data Availability

Pediatric dHCP data is publicly available. All code used in this study is publicly available: https://github.com/SrinivasanAnand/GNN_structural_connectivity.
